# Optimizing Sowing Date and Inter‐Row Spacing for Enhanced Yield and Yield Components of Mung Bean (*Vigna radiata* (L.) Wilczek) in the Rain‐Fed Agroecology of Western Ethiopia

**DOI:** 10.1155/sci5/1013462

**Published:** 2026-11-10

**Authors:** Birki Binalfew, Temesgen Fasika, Usman Mohammed Ali

**Affiliations:** ^1^ Department of Plant Sciences Faculty of Agriculture Wollega University Shambu Oromia Ethiopia wollegauniversity.edu.et

**Keywords:** harvest index, inter-row spacing, mung bean, phenology, seed yield, sowing date, *Vigna radiata*

## Abstract

Mung bean [*Vigna radiata* (L.) Wilczek] is a rapidly emerging, high‐value, short‐duration pulse crop in Ethiopia, offering significant nutritional and economic benefits. However, production in the rain‐fed agroecologies of Western Ethiopia is constrained by a lack of knowledge on optimal sowing windows and plant population density. This study was designed to identify the optimum combination of sowing date and inter‐row spacing that maximizes the yield and yield components of the Rasa mung bean variety. A field experiment was conducted under rain‐fed conditions during the 2024/25 main cropping season at the Uke site in the Guto Gida District of Western Ethiopia. The treatments were laid out in a 3 × 5 factorial arrangement using a Randomized Complete Block Design (RCBD) with three replications. The first factor was sowing date (July 20, July 30, and August 10), and the second was inter‐row spacing (20, 25, 30, 35, and 40 cm). Analysis of variance revealed that the main effect of sowing date was highly significant (*p* < 0.01) for nearly all parameters. Inter‐row spacing also had a significant (*p* < 0.05) to highly significant (*p* < 0.01) effect on key yield‐determining parameters. A highly significant (*p* < 0.01) interaction between sowing date and inter‐row spacing was observed for biomass yield, seed yield, straw yield, and harvest index. The combination of sowing on July 20 at a 25‐cm inter‐row spacing produced the maximum seed yield (2245.0 kg/ha^−1^), representing a 259.2% increase over the lowest‐yielding treatment (August 10 × 20 cm, 625.1 kg ha^−1^) and a 124.5% increase over the upper bound of typical farmer yields (1000 kg ha^−1^) in the region. Seed yield was most strongly correlated with leaf area index (*r* = 0.80, *p* < 0.01) and biological yield (*r* = 0.70, *p* < 0.01), indicating that yield in this environment is primarily source‐limited. The study provides evidence that the yield of the Rasa mung bean variety in the study area is profoundly influenced by an interactive effect of sowing date and plant spacing. To achieve optimal yield under rain‐fed conditions in Western Ethiopia and similar agroecologies, the combination of a July 20 sowing date and a 25‐cm inter‐row spacing is recommended, pending multiseason validation. These findings provide a research‐informed, location‐specific agronomic recommendation for mung bean farmers and extension agents.

## 1. Introduction

Global food and nutritional security continues to face unprecedented challenges from population growth, climate change, and resource degradation [1]. Recent analyses indicate that climate change, conflict, and economic shocks have compounded these challenges, with an estimated 733 million people facing hunger in 2023 [2]. The convergence of these stressors underscores the urgent need for resilient, short‐duration pulse crops that can stabilize food systems in marginal environments (Foyer et al. 2024).

In this context, grain legumes, or pulses, play an indispensable role due to their superior nutritional profile being rich in protein, essential minerals, and dietary fiber and their unique ability to fix atmospheric nitrogen, which enhances soil fertility and the sustainability of cereal‐based cropping systems [3–5]. Mung bean [*Vigna radiata* (L.) Wilczek], also known as green gram, is an annual pulse crop of primary importance in the semiarid and subtropical regions of Asia and, increasingly, Africa [6]. Originating from the Indian subcontinent, it has adapted remarkably well to diverse farming systems, from South and Southeast Asia to East Africa, including Ethiopia [7]. The crop is valued for its short growing cycle (60–80 days), drought tolerance, and versatility as a grain, vegetable (sprouts), and fodder source [8].

Nutritionally, mung bean is a powerhouse, with seed protein content ranging from 24% to 25%, complemented by carbohydrates (56.7%), fiber (4.1%), fat (1.3%), and essential minerals such as calcium and iron [9, 10]. This dense nutritional composition has earned it the colloquial designation of “affordable protein source for low‐income populations,” highlighting its critical role in providing affordable, high‐quality protein to low‐income populations [11]. Beyond direct human consumption, mung bean’s capacity to symbiotically fix atmospheric nitrogen (up to 42 kg/N/ha^−1^) makes it a vital component for maintaining soil health and reducing dependence on synthetic nitrogen fertilizers in subsequent crops [12]. Its deep root system also aids in accessing moisture from deeper soil layers, conferring a level of drought tolerance that is highly valuable under increasingly erratic rainfall patterns [13].

In Ethiopia, mung bean is a crop of growing agronomic and economic significance. It is the sixth commodity to be traded on the Ethiopian Commodity Exchange (ECX), following staples such as coffee, sesame, and maize [14], reflecting its rising national importance and market demand. As of 2023, mung bean remains among the top ten agricultural commodities traded on the ECX, reflecting sustained domestic and export demand [15]. The primary producing regions are concentrated in the lowlands and moisture‐stressed areas of the Amhara, Oromia, Benishangul‐Gumuz, and Southern Nations, Nationalities, and Peoples’ (SNNP) regions [7, 16]. The crop is particularly well‐suited to the lowland agroecology, which covers a vast area of the country, and fits well into farming systems involving intercropping, relay cropping with cereals using residual moisture, and sole cropping [17]. For smallholder farmers in these marginal environments, mung bean offers a dual advantage: a highly nutritious food source for household consumption and a valuable cash crop for generating income, particularly given the recent strong export market performance and high local prices [18].

Despite its immense potential, mung bean productivity in Ethiopia remains dismally low. National average yields hover around 800–1000 kg/ha^−1^ on farmers’ fields, compared to research station yields of up to 1800 kg ha^−1^ [19]. These figures represent national‐level estimates for rain‐fed smallholder systems [20]. In the specific lowland agroecology of Western Ethiopia, farmer yields are frequently at the lower end of this range due to the absence of location‐specific agronomic recommendations. This significant yield gap is attributable to a constellation of biotic and abiotic constraints. Key among these are the use of unimproved, low‐yielding landraces, suboptimal soil fertility management, and the prevalence of pests and diseases such as mung bean yellow mosaic virus (MYMV) and *Cercospora* leaf spot [21]. However, two of the most fundamental yet often overlooked constraints are inappropriate sowing dates (SDs) and suboptimal plant population densities (spacing). These are agronomic factors of the first order, as they profoundly influence the crop’s interaction with its growing environment throughout the entire life cycle.

SD is the single most critical nonmonetary input in crop production [22]. It determines the temporal window within which the crop’s entire growth and development must be completed, dictating the specific photothermal environment (temperature and day length regime) that the crop will experience during its vegetative and reproductive phases [23]. An optimal SD ensures that the crop’s critical growth stages particularly flowering and grain filling coincide with a favorable balance of solar radiation, temperature, and moisture availability, thereby maximizing the capture and conversion of resources into economic yield [24, 25]. Conversely, sowing too early or too late can expose the crop to terminal heat and drought stress, excessive rainfall during flowering (causing flower drop), or early frost, all leading to significant and often catastrophic yield reductions [26, 27]. A study by [28] demonstrated that delays in sowing shortened the vegetative phase and accelerated maturity, ultimately reducing the grain‐filling period (GP) and resultant seed yield (SY) in pulses [29].

Equally important is the management of plant population density through inter‐ and intrarow spacing. Spacing determines the level of interplant competition for growth‐limiting resources light, water, and nutrients [30, 31]. Canopy architecture directly governs radiation interception and radiation use efficiency (RUE); in mung bean, a stable extinction coefficient (*k* ≈ 0.68) and RUE of approximately 1.3 g/MJ^−1^ have been quantified, indicating that the leaf area index (LAI) is the primary driver of biomass accumulation under nonlimiting conditions [32]. In an excessively dense plant stand (narrow spacing), intense intraspecific competition leads to tall, spindly plants that are more susceptible to lodging and disease, with poor light interception in the lower canopy, reducing photosynthesis and pod formation [33]. The reduced air flow in dense canopies creates a humid microclimate conducive to fungal diseases. On the other hand, a very sparse plant stand (wide spacing) results in the underexploitation of available land area and resources. While individual plants may be more robust due to less competition, the yield per unit area is low because there are too few plants to collectively maximize resource capture [34]. The optimum spacing must therefore strike a delicate balance, achieving a plant population that maximizes canopy light interception (high LAI at the right time) while minimizing competitive stress and disease pressure [35].

The interaction between SD and plant spacing is of particular complexity. Environmental conditions vary greatly across a growing season. A specific plant spacing that is optimal under the cooler, wetter conditions of an early SD may become suboptimal under the hotter, drier conditions of a late SD, and vice versa [36]. For instance, under high‐temperature stress during late sowing, wider spacing might be necessary to reduce competition for limited soil moisture, while under early sowing conditions, narrower spacing might be needed to exploit the longer growing season and build sufficient biomass [37]. This intricate interplay must be unraveled through location‐specific factorial experiments.

In the specific context of the Uke area in the Guto Gida District of Western Ethiopia a predominantly lowland, rain‐fed agroecology mung bean is a relatively new, introduced crop [38]. Consequently, there is a profound lack of empirical, research‐based information on its best agronomic management practices. Farmers in this region often rely on generic recommendations or their own experience with other crops, leading to a haphazard selection of SDs and spacings [39]. The SD that is arbitrarily chosen, often as a secondary crop after the main cereal, frequently exposes the mung bean crop to strong terminal moisture stress. Similarly, inter‐row spacing (IRS) is often either too narrow, fostering intense competition, or excessively wide, wasting productive land. This knowledge gap is a primary driver for the low productivity witnessed in the area.

To date, no published study has systematically evaluated the SD × IRS interaction for mung bean in the rain‐fed lowland agroecology of Western Ethiopia. Previous agronomic work on mung bean in Ethiopia has focused on variety selection, fertilizer rates, or single‐factor spacing trials in other regions [40, 41], leaving the interactive effects of sowing time and plant density unquantified for this specific environment. This factorial experiment was therefore designed to address this critical gap.

Therefore, a well‐structured field experiment was designed with the following specific objectives:

1. To determine the optimum SD that maximizes SY and yield components of the Rasa mung bean variety in the study area.
2. To identify the most suitable IRS for enhanced growth and yield of mung bean.
3. To assess the interactive effect of SD and IRS on the phenology, growth, and yield performance of mung bean.
4. To provide a science‐based, location‐specific, integrated agronomic recommendation for mung bean farmers and extension agents in Western Ethiopia and similar rain‐fed lowland agroecologies.
5. To quantify the linear relationships between SY and key agronomic traits through correlation analysis, thereby identifying indirect selection criteria for yield improvement in this agroecology.

## 2. Materials and Methods

### 2.1. Description of the Experimental Site

The field experiment was carried out during the 2024/25 main cropping season under rain‐fed conditions at the Uke experimental site in the Guto Gida District of Western Oromia, Ethiopia. The site is geographically positioned at approximately 9°05′ N latitude and 36°55′ E longitude. It is located at an altitude ranging from 1300 to 1500 m above sea level (m.a.s.l.), which classifies it as a lowland agroecological zone. The location is approximately 40 km from the zonal capital, Nekemte town, and about 365 km west of Addis Ababa.

A comprehensive characterization of the experimental site was performed before planting. Soil texture was determined by the hydrometer method [42]. The measured particle‐size distribution was: sand 23.19%, silt 42.80%, and clay 33.68% (sum = 99.67%, with 0.33% unaccounted for due to organic matter and measurement error). According to the USDA textural triangle, this classifies as a loam soil. Selected chemical properties were as follows: pH 5.8 (1:2.5 soil:water), organic carbon 1.42%, total nitrogen 0.12%, available phosphorus (Olsen) 8.6 mg kg^−1^, and exchangeable potassium 0.24 cmol(+) kg^−1^. These analyses were performed at the Wollega University Soil Laboratory following standard procedures [42].

This textural classification is favorable for legume cultivation, offering a good balance of drainage and water‐holding capacity. Long‐term (1994–2023) mean annual rainfall is 1680 mm (range: 1200–2400 mm), with a monomodal distribution from May to September. The 2024/25 cropping season received 1542 mm of rainfall, with 612 mm falling during the mung bean–growing period (July–October). Mean monthly maximum and minimum temperatures during the growing period were 28.3°C and 15.1°C, respectively (data from the Uke Meteorological Station). The mean annual maximum and minimum temperatures are 31°C and 16°C, respectively, which is within the optimal range for mung bean growth and development. The farming system of the local community is a mixed crop–livestock production system, where crop residues are used to feed livestock and manure is used to fertilize croplands.

### 2.2. Experimental Materials

The genetic material used for this experiment was the mung bean variety “Rasa” (Accession No. N‐26/2010). Rasa was officially released in 2011 by the Melkassa Agricultural Research Center (MARC) of the Ethiopian Institute of Agricultural Research (EIAR). It is a widely recommended improved variety for the lowland agroecologies of the country due to its high yield potential and good adaptation. The specific agronomic attributes of the variety are detailed in Table 1.

**TABLE 1:** Description of the Mung bean variety “Rasa” (*N*‐26/2010) used for the experiment.

Agronomic trait	Description
Common name	Rasa
Pedigree name	N‐26/2010
Adaptation areas (m.a.s.l)	900–1570
Annual rainfall requirement (mm)	950 – 1150^∗^
Growth habit	Determinate
Days to 50% flowering	35–42
Days to 95% physiological maturity	65–80
Maturity group	Medium
Flower color	Yellow
Seed color	Green
Potential yield under research management (kg/ha^−1^)	1800
Typical yield under farmer management (kg/ha^−1^)	500–1000
Year of release	2011 G.C.
Seed rate (kg/ha^−1^)	40

^∗^Adapted from variety release documentation; field observations in Western Ethiopia suggest adequate performance at 900–1200 mm.

### 2.3. Experimental Treatments and Design

The experiment was structured as a two‐factor factorial arrangement, which allowed for the examination of both main effects and their interaction. The two factors were as follows:

1. SD: Three levels—July 20, July 30, and August 10. These dates were carefully selected to represent the early, mid, and late potential planting windows in the study area.
2. IRS: Five levels—20 cm, 25 cm, 30 cm, 35 cm, and 40 cm.

Intrarow spacing was maintained at a constant 10 cm for all treatments. This choice was made to isolate the effects of SD and IRS as the primary treatment factors. A 10‐cm intrarow spacing corresponds to the recommended practice for mung bean in Ethiopia [38] and yields plant densities of 25–50 plants m^−2^ across the IRS treatments, which brackets the range identified as optimal in previous spacing studies. This resulted in a total of 15 (3 × 5) unique treatment combinations. These treatments were laid out in a randomized complete block design (RCBD) with three replications, resulting in a total of 45 experimental plots. The RCBD was chosen to account for potential soil variability across the experimental field.

The gross plot size was 12 m^2^ (3 m width × 4 m length), and the net harvested plot size was 6 m^2^ (2 m × 3 m). A buffer zone of 0.5 m was maintained between adjacent plots, and a 1.5‐m‐wide alley was maintained between blocks to eliminate interference and ensure clear demarcation. The Rasa variety exhibits a determinate, compact growth habit (mean height ≈ 35 cm in this study), and a 0.5 m buffer was sufficient to prevent interplot interference. A wider 1.5‐m alley separated blocks.

### 2.4. Agronomic Management

All cultural and agronomic practices strictly followed the standard recommendations for mung bean production. The experimental field was prepared using a local ox‐drawn plow (*Gassoo*), with three passes to achieve a fine, level seedbed suitable for the small‐sized mung bean seeds. Manual bed preparation and leveling were done to ensure precise spacing and proper drainage.

A uniform seed rate of 40 kg ha^−1^ was used, and seeds were hand‐drilled at a depth of approximately 3 cm in rows spaced according to the designated treatment. At sowing, the recommended fertilizer rate of 100 kg/ha^−1^ NPS (a compound fertilizer containing 19% N, 38% P_2_O_5_, and 7% S) was applied uniformly to all plots. This rate follows the blanket recommendation for mung bean in Ethiopia [38] and was applied uniformly to ensure that nutrient availability did not confound treatment effects. No site‐specific soil test‐based fertilizer prescription was available for this location; this represents a limitation of the study and should be addressed in future research. The fertilizer was applied in a band a few centimeters away from the seed row to ensure maximum nutrient use efficiency and avoid salt injury.

Weeding was meticulously carried out twice to minimize any confounding effect of weed competition. The first hand‐weeding was conducted 20–25 days after sowing (DAS), and the second followed 40–45 DAS. Plant protection measures were implemented as guided by a regular pest and disease scouting schedule. Weekly scouting from emergence to physiological maturity recorded mean incidences of MYMV at 2.1% of plants and Cercospora leaf spot at 1.8% severity (0–5 scale). Pod borer damage affected 3.4% of pods. These levels were below economic thresholds, and no chemical control was applied.

### 2.5. Data Collection

A comprehensive dataset covering phenology, growth, and yield and its components was meticulously collected from the net plot area. The procedures for each parameter are described below.

#### 2.5.1. Phenological Parameters

❖ Days to 50% Emergence (DE): The number of days from sowing until 50% of the seedlings in each plot had visibly emerged above the soil surface. Emergence was recorded when the hypocotyl and cotyledons were visibly above the soil surface in 50% of the plants in a plot, assessed daily from 3 DAS.
❖ Days to 50% Flowering (DF): The number of days from sowing until 50% of the plants in each plot had at least one fully open flower. A plant was considered flowering when at least one fully open flower with visible petals was present. DF was recorded when 50% of plants in the net plot met this criterion.
❖ Days to 90% Physiological Maturity (DM): The number of days from sowing until 90% of the pods in the plot had turned yellowish‐brown to black, indicating physiological maturity.
❖ GP: The duration of the GP, calculated as the difference between days to DM and DF: GP = DM – DF.

#### 2.5.2. Growth Parameters

All growth parameters were measured on five randomly selected and tagged plants in the central rows of each net plot.

❖ Plant Height (PH): Measured in centimeters (cm) from the base of the plant at the soil surface to the tip of the main stem, at physiological maturity. The average of the five plants was recorded.
❖ Number of Primary Branches per Plant (NBP): The total number of primary branches arising from the main stem was counted on each of the five tagged plants, and the average was calculated.
❖ Number of Leaves per Plant (NLP): The total number of functional leaves was counted on each tagged plant at the 50% flowering stage.
❖ LAI: LAI, a measure of the canopy’s assimilatory surface area per unit of ground area, was nondestructively estimated at 30 DAS). Following the method established by Palanisamy and Gomez, the length (L) and maximum breadth (B) of the third fully expanded leaf from the top were measured for the five tagged plants [44]. The leaf area per plant was estimated using the formula: leaf area = *L* × B × *K*, where K is a constant factor (0.662) for green gram. The LAI was then calculated as the ratio of the total leaf area to ground area.

Equation (1): LAI calculation

(1) LAI: leaf area per plant x number of plants per plotground area of the plot.

#### 2.5.3. Yield and Yield Component Parameters

These parameters were measured at and after harvest from the net plot area to provide the most accurate yield estimate.

❖ Number of Pods per Plant (NPP): At full maturity, ten randomly selected plants from the central rows of each net plot were harvested, and the total number of pods on each plant was counted. The average was recorded as NPP.
❖ Pod Length (PL): Five randomly selected pods from each of the ten sample plants (50 pods per plot) were measured from the tip to the base in centimeters, and the mean was calculated.
❖ Number of Seeds per Pod (NSP): The same 50 pods used for measuring PL were threshed, and the number of healthy seeds per pod was counted and averaged.
❖ Thousand Seed Weight (TSW) (g): One thousand clean, sun‐dried seeds from the net plot yield were counted using a precision seed counter and weighed on a calibrated electronic balance at a moisture content adjusted to 12.5%. Seed moisture was determined using a calibrated moisture meter (Sartorius MA35) and adjusted to 12.5% following the ISTA rules [45].
❖ Biological Yield (BY) (kg/ha^−1^): The total above‐ground biomass from the entire net plot area was harvested, bundled, and sun‐dried until a constant weight was achieved. The total dry weight was recorded in kilograms per plot and then converted to kilograms per hectare.
❖ SY (kg/ha^−1^): The dried bundles were threshed manually. The seeds were cleaned, sun‐dried to uniform moisture content of 12.5%, and weighed. The plot‐level seed weight was then converted to SY in kg ha^−1^.
❖ Straw Yield (STY) (kg/ha^−1^): This was calculated for each plot as the difference between the BY and the SY: STY = BY – SY.
❖ Harvest Index (HI): This is a measure of the crop’s efficiency in partitioning photosynthates to the economic product (seed). It is a unitless ratio, expressed as a percentage, calculated as follows:
❖ Equation (2): HI (%) calculation
(2) HI%seed Yield kg ha−1biological yieldkg ha−1×100.

### 2.6. Statistical Data Analysis

The integrity of all collected data was first assessed using the assumption tests of homogeneity of variance (Levene’s test) and normality of residuals (Shapiro–Wilk test) to ensure the validity of the subsequent ANOVA. Data were also screened for outliers using studentized residuals (threshold |r| > 3); no outliers were removed as all residuals fell within acceptable limits, and missing data were absent in this experiment. All parameters were subjected to a two‐way analysis of variance (ANOVA) using the generalized linear model (GLM) procedure in R statistical software Version 4.2 [46]. The model included the effects of SD, IRS, and their interaction as fixed factors, with replication as a random factor.

The ANOVA model was as follows: Yijk = *μ* + ρk + αi + βj + (αβ)ij + εijk,

where *μ* is the overall mean, ρk is the block effect, αi is the SD effect, βj is the IRS effect, (αβ)ij is the interaction, and εijk is the residual error.

Where significant differences (*p* ≤ 0.05) were detected by the F‐test, treatment means were separated using Tukey’s honestly significant difference (HSD) test at *α* = 0.05, which controls the experiment‐wise error rate and is more conservative than Fisher’s least significant difference (LSD) test, particularly given the presence of 15 treatment combinations where LSD is known to inflate the probability of Type I errors [47]. This approach follows the recommendation of Gomez and Gomez [48] for factorial experiments with multiple treatment combinations.

Furthermore, Pearson’s bivariate correlation analysis was performed on all measured traits to quantify the strength and direction of the linear relationships between them, with a specific focus on identifying the key determinants of SY. The significance of all correlation coefficients was tested at the 5% and 1% probability levels. It should be noted that these correlation coefficients describe linear associations among treatment means and do not imply causation, as several traits are physiologically interdependent and multicollinearity among predictors is likely. Path analysis or multiple regression on plot‐level data would be required to partition direct and indirect effects.

## 3. Results

### 3.1. ANOVA

The comprehensive ANOVA results for all measured phenological, growth, yield, and yield component traits are presented in Table 2. The SD demonstrated a highly significant (*p* < 0.01) main effect on almost all parameters, including number of DF, days to 90% maturity, GP, PH, NLP, number of branches per plant, PL, NPP, NSP, BY, SY, and STY. The effect of SD was only significant at *p* < 0.05 for the LAI, and nonsignificant (*p* > 0.05) for DE, thousand seed weight, and HI. This underlines the overarching influence of planting time on the crop’s entire ontogeny and productivity. The relatively high coefficient of variation (CV) (10.11%) for DE reflects a minor plot‐to‐plot variation in soil moisture at sowing, likely due to microtopographic differences. Since emergence differences were not statistically significant among treatments, this variability did not bias subsequent treatment comparisons.

**TABLE 2:** Mean squares from the analysis of variance (ANOVA) for phenological, growth, yield, and yield component parameters of mung bean as affected by sowing date (SD), inter‐row spacing (IRS), and their interaction (SD × IRS).

Source of variation	df	Days to 50% emergence	Days to 50% flowering	Days to 90% maturity	Grain filling period	Plant height (cm)	No. of leaves per plant	No. of branches per plant	Leaf area index (30 DAS)	Pod length (cm)	No. of pods per plant	No. of seeds per pod	Thousand seed weight (g)	Biological yield (kg ha^−1^)	Seed yield (kg ha^−1^)	Straw yield (kg ha^−1^)	Harvest index
Sowing date (SD)	2	0.82 ns	807.62^∗∗^	421.62^∗∗^	63.26^∗∗^	501.71^∗∗^	99.64^∗∗^	33.76^∗∗^	0.013^∗^	31.82^∗∗^	42.68^∗∗^	30.27^∗∗^	100.61 ns	31,379,064.0^∗∗^	2,252,764.7^∗∗^	18,399,742.6^∗∗^	0.0026 ns
Inter‐row spacing (IRS)	4	1.35^∗^	3.30 ns	3.94 ns	5.86 ns	10.29 ns	3.89^∗^	3.25 ns	0.067^∗∗^	1.14 ns	0.98 ns	0.58 ns	53.34 ns	15,347,713.7^∗∗^	449,466.6^∗∗^	12,906,957.8^∗∗^	0.0244^∗∗^
SD × IRS	8	1.07^∗^	2.53 ns	5.34 ns	2.51 ns	5.47 ns	2.30 ns	1.87 ns	0.002 ns	1.27 ns	1.00 ns	0.63 ns	44.67 ns	5,165,130.3^∗∗^	219,852.9^∗∗^	3,742,742.7^∗∗^	0.0066^∗∗^
Residual error	28	0.48	3.18	3.23	5.65	10.87	1.09	1.016	0.002	1.77	2.26	0.98	35.43	572,592.6	43,471.7	422,107.2	0.0014
CV (%)	—	10.11	3.91	2.20	6.59	9.82	13.25	19.38	17.94	11.80	12.45	10.81	9.52	16.15	17.30	18.76	14.26

N*ote:* ns = nonsignificant (*p* > 0.05); total df = 44; block df = 2.

Abbreviation: CV = coefficient of variation.

^∗^significant at *p* < 0.05.

^∗∗^significant at *p* < 0.01.

The main effect of IRS was also highly significant (*p* < 0.01) for LAI, BY, SY, STY, and HI. It was significant (*p* < 0.05) for DE and NLP. For the remaining parameters DF, days to 90% maturity, GP, PH, number of branches per plant, PL, pod number per plant, NSP, and thousand seed weight, the effect of IRS was nonsignificant. This pattern indicates that plant spacing primarily affects yield and its components through its influence on canopy development, light interception (as captured by LAI), and the overall efficiency of resource use per unit area.

Crucially, the interaction effect of SD and IRS (SD × IRS) was highly significant (*p* < 0.01) for four key agronomic and economic parameters: BY, SY, STY, and HI. It was also significant (*p* < 0.05) for DE.

This profound interaction confirms that the effect of plant spacing on the ultimate productivity of mung bean cannot be generalized but is contingent upon the date of sowing. It validates the experimental design’s factorial structure and necessitates a focused analysis of the interaction means rather than merely the main effects for these critical traits. The CVs for most measured traits were within an acceptable range (10%–20%), indicating good precision in experimental management and data collection.

### 3.2. Effect of SD on Phenology, Growth, and Yield Components

#### 3.2.1. Phenological Parameters

The main effect of SD on the phenological development of mung bean was profound and highly significant (*p* < 0.01) (Table 3). DE did not differ significantly among the SDs, remaining relatively constant across the three dates (data not shown in main effect tables). This confirms that soil moisture and temperature conditions at the time of sowing were sufficient for uniform germination for all three planting events.

**TABLE 3:** Main effect of sowing date on phenological, growth, yield, and yield component traits of the Rasa mung bean variety.

Sowing date	DF (days)	DM (days)	GP (days)	PH (cm)	NBP	NLP	LAI	PL (cm)	NPP	NSP
July 20	53.13^a^	87.00^a^	33.86^c^	30.84^b^	5.00^b^	7.40^b^	0.22^b^	11.49^b^	12.78^b^	10.56^b^
July 30	45.33^b^	81.93^b^	36.40^b^	40.22^a^	6.79^a^	10.64^a^	0.28^a^	12.64^a^	14.21^a^	11.89^a^
August 10	38.46^c^	76.40^c^	37.93^a^	29.68^c^	3.81^c^	5.58^c^	0.27^a^	9.75^c^	9.28^c^	9.36^c^
Tukey HSD (α = 0.05)	1.33	1.34	1.77	2.46	0.75	0.78	0.03	0.99	1.13	0.70
CV (%)	3.91	2.20	6.59	9.82	19.37	13.25	17.94	11.80	12.45	10.81

*Note:* Means within the same column followed by different superscript letters are significantly different at *p* ≤ 0.05. DF = days to 50% flowering, DM = days to 90% maturity, GP = grain‐filling period.

Abbreviations: LAI = leaf area index, NBP = number of branches per plant, NLP = number of leaves per plant, NPP = number of pods per plant, NSP = number of seeds per pod, PH = plant height, PL = pod length.

In contrast, DF showed a substantial response. The duration from sowing to flowering progressively and significantly shortened with a delay in sowing. The early sown crop (July 20) took the longest to flower, averaging 53.13 days. This period decreased by 7.8 days–45.33 days for the July 30 sowing and further declined to only 38.46 days for the late sowing on August 10. The difference between the earliest and latest sowing was a substantial 14.67 days, a reduction primarily driven by increasing temperature and a shifting photoperiod that accelerated phenological development in the later‐planted crop. In contrast to flowering, DE did not differ significantly among SDs, indicating that soil moisture and temperature at sowing were sufficient for uniform germination across all three planting dates. This is consistent with the onset of the main rains in early July at this location, which ensured adequate moisture for germination regardless of SD.

A highly analogous trend was observed for DM. The early sown (July 20) crop matured in 87.00 days, the mid‐sown (July 30) crop in 81.93 days, and the late‐sown (August 10) crop in just 76.40 days. The 10.6‐day earlier maturity of the late‐sown crop is of important practical significance, as it reduces the crop’s total exposure time to potential end‐of‐season stresses. The late‐sown crop entered its postflowering phase in late September and October, when mean daily temperatures declined from 24.8°C (during vegetative phase) to 21.2°C, and rainfall ceased after mid‐October.

The GP, which is the most critical phase for SY determination, exhibited the exact opposite trend. The longest GP of 37.93 days was recorded for the late‐sown (August 10) crop. The mid‐sown crop had an intermediate GP of 36.40 days, while the early sown crop had the shortest GP of only 33.86 days. This indicates that although late sowing accelerates the onset of flowering and maturation, the actual period from flowering to maturity, the grain‐filling phase is extended. This has significant implications for seed filling and quality and is a critical finding for understanding the crop’s response to the thermal regime.

#### 3.2.2. Growth Parameters

All measured growth parameters were significantly (*p* < 0.05) influenced by SD (Table 3). In contrast to the unidirectional linear trend seen in phenology, growth parameters and yield components exhibited a distinct parabolic, or mid‐optimum, response. The highest values for PH (40.22 cm), NBP (6.79), and NLP (10.64) were consistently achieved with the mid‐July 30 SD. The early (July 20) SD showed intermediate values for these parameters. The late (August 10) SD resulted in significantly the poorest performance, with the most stunted plant growth: PH (29.68 cm), branches per plant (3.81), and leaves per plant (5.58). The LAI followed a similar pattern, with the July 30 sowing recording the maximum LAI of 0.28, which was statistically on par with the August 10 sowing (0.27), both being superior to the July 20 sowing (0.22).

This pattern strongly suggests that the environmental conditions (specifically the interplay of temperature and moisture) prevailing during the vegetative phase of the July 30 sowing were most conducive to robust plant growth. The July 20 sowing, while having a longer vegetative phase, likely experienced cooler initial temperatures that limited growth rate, whereas the August 10 sowing, though warmer, had a drastically shortened vegetative period, preventing substantial biomass accumulation.

#### 3.2.3. Yield and Yield Components

The NPP, PL, and NSP all displayed the identical parabolic response to SD, with the July 30 sowing outperforming the others (Table 3). The July 30 sowing produced an average of 14.21 pods per plant, a mean PL of 12.64 cm, and 11.89 seeds per pod. These were followed by the July 20 sowing. The late August 10 sowing was significantly inferior across all three traits, with mean values of 9.28 pods per plant, 9.75 cm PL, and 9.36 seeds per pod. The substantial reduction in the pod number (a 34.7% decrease compared to the July 30 sowing) is a primary agronomic cause for the yield penalty associated with delayed planting.

### 3.3. Effect of IRS on Growth Parameters

The main effect of IRS was significant for two key canopy‐related growth parameters: NLP and LA. As detailed in Table 4, the response of these two traits to spacing was distinctly different.

**TABLE 4:** Main effect of inter‐row spacing on growth parameters of mung bean.

Inter‐row spacing (cm)	Number of leaves per plant (NLP)	Leaf area index (LAI) at 30 DAS
20	7.51^b^	0.37^a^
25	8.58^a^	0.32^b^
30	8.28^ab^	0.22^c^
35	8.04^ab^	0.20^cd^
40	6.96^c^	0.17^d^
Tukey HSD (*α* = 0.05)	1.00	0.04
CV (%)	13.25	17.94
Mean	7.89	0.26

*Note:* Means within the same column followed by different superscript letters are significantly different at *p* ≤ 0.05.

Abbreviation: DAS = days after sowing.

The NLP, a measure of individual plant vigor, showed a curvilinear response to increasing IRS. It peaked at a spacing of 25 cm (8.58 leaves), followed by 30 cm (8.28) and 35 cm (8.04), all of which were statistically similar. The narrowest (20 cm) and widest (40 cm) spacings resulted in the lowest NLP (7.51 and 6.96, respectively). The reduction in the leaf number at the 20 cm spacing can be attributed to intense intrarow competition for resources, which stifles the growth of individual plants. The significant decline at the 40‐cm spacing is likely a feedback response. The reduced leaf number at 40‐cm spacing may reflect a shift in dry matter allocation toward stem thickening or root development under low‐density conditions, a response documented in other grain legumes [30]. However, this hypothesis was not directly tested in the present study.

Conversely, the LAI, which is a canopy‐level attribute, showed a strong, negative, linear relationship with increasing IRS. The highest LAI value of 0.37 was obtained at the narrowest spacing of 20 cm. The LAI then progressively and significantly declined to 0.32 (25 cm), 0.22 (30 cm), and 0.20 (35 cm) and reached its lowest point of 0.17 at the widest 40‐cm spacing. This is a clear and expected result, demonstrating that the higher plant population density per unit area in narrowly spaced rows creates a more extensive leaf surface area per unit of ground, which is crucial for maximizing light capture early in the season.

The relatively low LAI values reported here reflect the early measurement time (30 DAS) and the nondestructive estimation method based on linear leaf dimensions (*L* × *B* × 0.662). These values should be interpreted as relative indices for treatment comparison rather than absolute canopy leaf area. Destructive sampling or ceptometer‐based measurements would likely yield higher absolute values.

### 3.4. Interaction Effect of SD and IRS on Key Yield Parameters

The highly significant (*p* < 0.01) interaction between SD and IRS on SY, BY, STY, and HI (Table 2) is the central and most important finding of this study. It indicates that the optimum spacing for a given SD is specific, and a universal recommendation cannot be made. The following sections dissect these interactions.

#### 3.4.1. Biological and STY

The BY (total above‐ground dry matter), which represents the total system productivity, ranged widely from 2373.3 kg/ha^−1^–8395.7 kg/ha^−1^ (Table 5). The highest BY was recorded for the July 30 sowing at a 20‐cm IRS (8395.7 kg ha^−1^), which was statistically at par with the July 20 sowing at 25‐cm spacing (8191.2 kg/ha^−1^). This suggests that both early planting with a moderate spacing and mid‐season planting with high density can maximize biomass accumulation.

**TABLE 5:** Interaction effect of sowing date and inter‐row spacing on biological yield (BY, kg/ha^−1^) and straw yield (STY, kg/ha^−1^) of mung bean.

Inter‐row spacing (cm)	Biological yield (kg/ha^−1^)			Straw yield (kg/ha^−1^)		
	July 20	July 30	Aug 10	July 20	July 30	Aug 10
20	6565.9^b^	8395.7^a^	3366.7^ef^	5016.1^c^	7085.5^a^	2741.6^g^
25	8191.2^a^	7157.3^b^	2526.7^f^	5945.9^b^	5630.8^bc^	1871.6ʰ
30	5367.0^cd^	4372.0^e^	3384.0^ef^	3855.3^ef^	2949.4^z^	2140.9^g^ʰ
35	3657.3^z^	4412.9^de^	3426.2^ef^	2437.5^g^	3168.6^fz^	2761.4^z^ʰ
40	3792.9^ef^	3293.0^f^	2373.3ʰ	2685.2^g^	2228.2^g^	1443.5^i^
Tukey HSD (*α* = 0.05)	BY: 1222.6	STY: 1052.8				
CV (%)	BY: 16.15	STY: 18.76				

*Note:* Means within a parameter (BY or STY) followed by different superscript letters are significantly different at *p* ≤ 0.05.

In stark contrast, the lowest BY (2373.3 kg/ha^−1^), a 3.5‐fold reduction from the maximum, was obtained from the combination of the latest SD (August 10) and the widest spacing (40 cm). All late‐sowing treatments, irrespective of spacing, yielded lower biomass compared to their earlier‐sown counterparts, highlighting the overwhelming negative effect of delayed sowing on overall crop growth. However, the impact of spacing within each SD was substantial. For the July 20 sowing, biomass increased from 6565.9 kg ha^−1^ at 20 cm to a peak of 8191.2 kg ha^−1^ at 25 cm before declining at wider spacings. For the July 30 sowing, biomass was highest at the narrowest 20‐cm spacing and declined consistently as row width increased.

Notably, the highest BY (8395.7 kg/ha^−1^) was obtained from July 30 × 20 cm, whereas the highest SY (2245.0 kg/ha^−1^) came from July 20 × 25 cm. This divergence indicates that maximum biomass accumulation does not necessarily coincide with maximum partitioning efficiency and that the July 20 × 25 cm combination achieved a superior balance between total dry matter production and HI (27.4%).

The pattern for STY (Table 5) closely mirrored that of BY, as straw constitutes the major portion of the total biomass. The maximum STY of 7085.5 kg/ha^−1^ was obtained from the July 30 sowing at 20‐cm IRS, which was statistically similar to the July 20 sowing at 25‐cm spacing (5945.9 kg/ha^−1^). The lowest STY (1443.5 kg/ha^−1^) was from the late‐sown, widest‐spaced crop. This consistent pattern reinforces the conclusion that early to mid‐season planting and moderate‐to‐narrow spacing are essential for maximizing total biomass and, therefore, the vegetation cover and potential for organic matter restitution.

#### 3.4.2. SY and HI

The ultimate economic goal of mung bean production is SY, and its optimization was the primary objective of this experiment. The interaction effect on SY was highly significant, with values ranging from a minimum of 625.1 kg/ha^−1^ to a maximum of 2245.0 kg/ha^−1^ (Table 6).

**TABLE 6:** Interaction effect of sowing date and inter‐row spacing on seed yield (SY, kg/ha^−1^) and harvest index (HI) of mung bean.

Inter‐row spacing (cm)	Seed yield (kg ha^−1^)			Harvest index (HI) (%)		
	July 20	July 30	Aug 10	July 20	July 30	Aug 10
20	1549.8^b^	1310.1^c^	625.1^e^	23.5^ef^	15.6ʰ	18.6^z^
25	2245.0^a^	1526.5^b^	655.1^e^	27.4^d^	21.4^z^	25.9^de^
30	1511.7^bc^	1422.6^c^	1242.3^d^	28.1^d^	32.5^ab^	36.1^a^
35	1219.8^d^	1244.4^d^	664.8^e^	33.3^ab^	29.1^cd^	19.4^z^
40	1107.7^e^	1064.8^e^	687.2^e^	29.1^cd^	32.3^bc^	28.3^d^
Tukey HSD (*α* = 0.05)	SY: 348.74	HI: 0.06				
CV (%)	SY: 17.30	HI: 14.26				

*Note:* Means within a parameter (SY or HI) followed by different superscript letters are significantly different at *p* ≤ 0.05.

The clear winner, producing the maximum SY, was the treatment combination of the July 20 SD with a 25‐cm IRS (2245.0 kg/ha^−1^). This yield was not only the highest but also statistically superior to all other treatment combinations. The second‐highest yield (1549.8 kg/ha^−1^) was obtained from the July 20 sowing at 20‐cm spacing, demonstrating the synergy between early planting and narrow‐to‐moderate row spacing.

This optimum treatment (July 20 × 25 cm) outperformed the worst‐performing treatment (Aug 10 × 20 cm) by a staggering 1619.9 kg/ha^−1^ (259.2% increases). It also substantially exceeded the yield potential of the Rasa variety in farmers’ fields (500–1000 kg/ha^−1^) and even surpassed the upper ceiling of its research field yield (1800 kg/ha^−1^), as documented in the variety’s release description (Table 1). This clearly demonstrates the immense yield gains achievable simply by optimizing these low‐cost agronomic inputs.

The HI, an indicator of partitioning efficiency, ranged from 15% to 36%. The highest HI (36%) was achieved with the August 10 sowing at 30‐cm spacing, which is a classic stress‐avoidance response: A late‐planted crop with a limited canopy often allocates a greater fraction of its limited biomass to seeds as a survival mechanism. The significant interaction highlights that the physiological efficiency of converting total biomass into economic yield is jointly and profoundly controlled by sowing time and population density.

The high HI under late sowing at 30 cm (36.1%) should not be interpreted as agronomically desirable, as it was achieved under severely constrained biomass production. As noted by Hay [49], a high HI in stress environments often reflects reproductive survival mechanisms rather than optimal resource‐use efficiency.

### 3.5. Correlation Between SY and Other Agronomic Traits

To understand the physiological basis for the yield differences, a Pearson correlation analysis was conducted between SY and all other measured traits (Table 7). The analysis revealed highly significant (*p* < 0.01) positive correlations between SY and several key traits, providing a clear biological roadmap for yield improvement.

**TABLE 7:** Pearson correlation coefficients (*r*) between pairwise combinations of traits in mung bean.

Traits	SY	By	STY	HI	LAI	NPP	NSP	NBP	DF	DM	GP
SY	1										
BY	0.70^∗∗^	1									
STY	0.65^∗∗^	0.98^∗∗^	1								
HI	0.70^∗∗^	0.42^∗^	0.56^∗^	1							
LAI	0.80^∗∗^	0.40^∗^	0.45^∗^	0.52^∗^	1						
NPP	0.46^∗^	0.45^∗∗^	0.42^∗^	0.04^ns^	0.02^ns^	1					
NSP	0.62^∗∗^	0.35^∗^	0.33^ns^	0.02^ns^	0.07^ns^	0.67^∗∗^	1				
NBP	0.52^∗^	0.48^∗^	0.44^∗^	0.10^ns^	0.26^ns^	0.69^∗^	0.53^∗^	1			
DF	0.60^∗∗^	−0.48^∗^	−0.41^∗^	0.09^ns^	0.22^ns^	0.56^∗∗^	0.35^ns^	0.34^ns^	1		
DM	0.57^∗∗^	−0.39^∗^	−0.32^ns^	0.20^ns^	0.29^ns^	−0.50^∗^	0.37^ns^	0.24^ns^	0.91^∗∗^	1	
GP	0.36^∗^	0.40^∗^	0.37^∗^	0.11^ns^	0.00^ns^	0.35^ns^	0.09^ns^	0.34^ns^	0.70^∗∗^	−0.37^∗^	1

*Note:* STY = straw yield; DF = days to 50% flowering; DM = days to 90% maturity; GP = grain‐filling period. Note: Correlations are based on treatment means (*n* = 15); caution is warranted in interpreting these coefficients, as they may be inflated relative to correlations based on individual plot data. These correlation coefficients describe linear associations among treatment means and do not imply causation. Several traits are physiologically interdependent, and multicollinearity among predictors is likely. Path analysis or multiple regression on plot‐level data would be required to partition direct and indirect effects.

Abbreviations: BY = biological yield, HI = harvest index, LAI = leaf area index, NBP = number of branches per plant, NPP = number of pods per plant, ns = nonsignificant, NSP = number of seeds per pod, SY = seed yield.

^∗^Significant at *p* < 0.05.

^∗∗^Highly significant at *p* < 0.01.

SY was most strongly and positively correlated with LAI (*r* = 0.80^∗∗^) and BY (*r* = 0.70^∗∗^). This underscores the fundamental principle that high economic yield is a product of high total biomass and a well‐developed canopy capable of capturing solar radiation. The strong positive correlation with STY (*r* = 0.65^∗∗^) further reinforces the link between overall vegetative vigor and productivity.

At the component level, SY showed strong positive associations with NSP (*r* = 0.62^∗∗^), DF (*r* = 0.60^∗∗^), days to 90% maturity (*r* = 0.57^∗∗^), number of branches per plant (*r* = 0.52^∗^), NLP (*r* = 0.46^∗^), and NPP (*r* = 0.46^∗^). These correlations collectively indicate that treatments (like the July 20 × 25 cm combination) that prolonged the vegetative and reproductive phases and enhanced the plant’s structural framework (branches and leaves) created a larger “sink” for photosynthates, resulting in more and better‐filled pods and seeds, and ultimately higher SY. The positive correlation with GP (*r* = 0.36^∗^) suggests a more nuanced role, where a moderate extension of the grain‐filling duration under favorable early sown conditions can be beneficial.

Conversely, traits like PH (*r* = 0.28 ns), pod length (*r* = 0.30 ns), and DE (*r* = −0.06 ns) showed no significant linear correlation with SY, indicating that these attributes are less reliable as indirect selection criteria for yield improvement in this specific context.

## 4. Discussion

### 4.1. Introduction and Study Context

This single‐season study (2024/25) was designed to address a critical knowledge gap regarding the agronomic management of the recently introduced mung bean crop in the lowland, rain‐fed agroecosystem of Western Ethiopia. The findings provide evidence that both SD and IRS are powerful, nonmonetary determinants of yield, and their significant interaction necessitates an integrated management approach.

### 4.2. Influence of SD on Crop Phenology

The progressive shortening of the vegetative period (days to flowering and maturity) with delayed sowing is a well‐documented physiological response in short‐day plants. As sowing is delayed from late July to mid‐August, the crop is exposed to higher average daily temperatures and, in many photoperiod‐sensitive genotypes, a shortening day length [48]. Both factors trigger a faster transition from the vegetative to the reproductive phase, curtailing the time available for biomass accumulation [23]. This aligns with the findings of Palsaniya et al. [22], who noted that thermal time accumulation is faster for later‐sown mung bean, accelerating the onset of flowering [22]. The reduction in the length of the vegetative phase for the August 10 crop observed in our study is a direct consequence of this increased rate of thermal unit accumulation.

The temperature and moisture gradients recorded during the experimental season provide quantitative support for this phenological acceleration. Mean daily temperature during the vegetative phase was 21.5°C for the July 20 sowing, 23.1°C for July 30, and 24.8°C for August 10, based on in‐field data loggers (HOBO UX100‐003). Cumulative rainfall from sowing to 50% flowering was 285 mm (July 20), 198 mm (July 30), and 87 mm (August 10). These temperature and moisture gradients explain the progressive acceleration of phenological development with delayed sowing, as higher temperatures increased the rate of thermal unit accumulation while declining moisture availability further shortened the vegetative phase.

Perhaps the most insightful phenological finding is that while delayed sowing shortened the total crop duration, it significantly extended the GP. This is not contradictory but rather illustrates a complex plant–environment interaction. The extended GP under late sowing (37.93 days vs. 33.86 days for early sowing) occurred under declining temperatures (mean 21.2°C in October vs. 23.8°C in August) and terminal moisture stress (rainfall ceased by mid‐October). While cooler temperatures can prolong grain‐filling duration, the concurrent water deficit likely negated any potential benefit by accelerating leaf senescence and reducing assimilate supply. The study by Eshanee et al. [50] on snow pea corroborates such a response, showing that the reproductive growth rate is highly sensitive to temperature, and a late shift to a cooler thermal regime can decelerate seed development. Under the terminal drought stress conditions typical of rain‐fed Ethiopian lowlands, any benefit from a longer GP is often negated by the accelerated leaf senescence and reduced photosynthetic capacity induced by moisture scarcity, a key factor in the yield reduction of the late‐sown crop.

### 4.3. Effect of SD and IRS on Growth and Canopy Development

The strong vegetative growth observed in the July 30 sowing (Table 3) indicates that this planting window provided the optimal photothermal balance for the entire life cycle. The July 20 crop, though benefiting from a longer total duration, likely experienced a suboptimal early growth rate due to lower temperatures. Conversely, the August 10 crop, while experiencing high early temperatures, had a vegetative phase too short to build the necessary plant structure (height, branches, leaves) to support high yields [39]. These findings are in complete agreement with the results of Kumar and Kumawat [26], who reported significantly reduced PH and branching in late‐sown mung bean in the Eastern Himalayas.

The analysis of canopy characteristics under varying spacing (Table 4) revealed the classic trade‐off between individual plant performance and community‐level resource capture. At a narrow 20‐cm spacing, the individual plant leaf number was reduced due to intense competition, but the LAI, a community trait, was at its maximum. A high LAI is critical for early season light interception, suppressing weeds and maximizing the potential for photosynthesis [35]. The radiation extinction coefficient (*k*) for mung bean canopies has been estimated at approximately 0.68, and RUE at 1.3 g/MJ^−1^, providing a quantitative basis for interpreting the LAI differences observed here [32]. The progressive and significant decline in LAI with wider row spacing explains the associated decrease in BY per unit area. This confirms the fundamental crop physiological principle that maximizing productivity requires an early developing, dense canopy that captures most of the incident solar radiation throughout the growth cycle [33]. The study of Zhou et al. [35] on soybean also found that narrow row spacing led to higher light interception, demonstrating the universality of this principle across grain legumes. Recent work on common bean and other grain legumes has confirmed that narrow row spacing consistently increases radiation interception and biomass accumulation, although the magnitude of yield response depends on seasonal moisture availability [31].

### 4.4. The Crucial Interaction: Optimizing SY and HI

The highly significant interaction effect on SY (Table 6) is the most critical finding of this study. It confirms our initial hypothesis that the optimum spacing is not static but is dependent on the date of sowing. The top‐performing treatment, July 20 sowing with 25‐cm IRS, produced a notable 2245.0 kg/ha^−1^, a yield that exceeds the previously documented yield ceiling for this variety [19]. This synergistic combination worked because it allowed the crop to

1. Capitalize on the favorable early season moisture and moderate temperatures for a prolonged vegetative period to build a robust structure (good PH and branching, as per the July 20 main effect).
2. Achieve a sufficiently high plant population (via a moderate 25‐cm row) to maximize LAI and thus BY [35].
3. Avoid the intense intrarow competition at the 20‐cm spacing, which would have been amplified by the long growing season and led to reduced pod and seed set per plant [18].

This combination achieved a functional equilibrium between interplant competition and canopy resource capture: the 25‐cm row spacing allowed sufficient plant density to achieve a high LAI (0.32) and BY, while avoiding the intense intraspecific competition at 20 cm that reduced pods per plant and seeds per pod.

The dramatic yield decline in the late‐sown (August 10) crop, even at the narrowest spacing, was inevitable. The combination of a severely restricted vegetative phase, leading to low source potential (low biomass), and the onset of terminal drought stress during the extended GP created an unfavorable convergence of factors for crop failure. This explains the low yields of 625–687 kg/ha^−1^, which are below even the national average for farmers’ fields [19]. These results starkly illustrate the high risk associated with delayed sowing in this rain‐fed environment.

The rainfall data provide strong support for this interpretation. Cumulative rainfall from sowing to maturity was 612 mm for July 20, 498 mm for July 30, and 287 mm for August 10. The late‐sown crop experienced terminal drought stress during the pod‐filling phase, as no effective rainfall occurred after mid‐October, which coincided with the GP of the August 10 sowing. The August 10 sowing received only 287 mm of rainfall from sowing to maturity, compared with 612 mm for the July 20 sowing. Furthermore, the GP of the late‐sown crop coincided with the cessation of rains in mid‐October, subjecting the crop to terminal drought stress during the critical seed‐filling phase.

The HI findings further enrich this interpretation. The highest HI (36.1%) was recorded for the August 10 × 30 cm treatment. This is a compensatory, stress‐avoidance mechanism. A late‐sown plant, with limited vegetative growth and facing terminal stress, will often prioritize reproductive development and remobilize assimilates from stems and leaves to the developing seeds, thereby increasing the partitioning efficiency (HI) as a survival strategy [51]. However, the high HI observed under late sowing (36.1%) is a compensatory response to stress and should not be interpreted as a desirable agronomic outcome. As emphasized by Hay [49] and Sinclair [52], a high HI under low‐yielding conditions reflects survival‐driven partitioning rather than efficient resource use. The primary pathway to high SY in this environment is the maximization of BY through optimal early sowing and plant density. While a high HI is desirable, our results clearly show that for rain‐fed systems, the path to high SY is to first maximize total BY through optimal early sowing and plant density, which the July 20 × 25 cm combination achieved. The strong positive correlation between SY and BY (*r* = 0.70) and LAI (*r* = 0.80) in Table 7 confirms that in this environment, yield is source‐limited; it depends first and foremost on having a large, photosynthetically active canopy to drive dry matter production.

### 4.5. Divergence Between Maximum Vegetative Growth and Maximum SY

A notable feature of these results is that the treatment producing the greatest vegetative growth (July 30 sowing; PH 40.22 cm, branches 6.79, leaves 10.64) did not produce the highest SY. The July 20 sowing, despite intermediate vegetative values, achieved a superior SY when combined with 25‐cm spacing. This apparent paradox can be explained by differences in biomass partitioning: the July 30 crop, while accumulating more total dry matter (BY 8395.7 kg/ha^−1^ at 20 cm), partitioned a smaller fraction to grain (HI 15.6% at 20 cm) compared with the July 20 × 25 cm treatment (HI 27.4%). Thus, high vegetative vigor alone is insufficient for high yield; the critical factor is the coordinated optimization of both biomass production and partitioning efficiency. These findings are consistent with the concept of source–sink balance.

### 4.6. Comparison With Other Ethiopian and Tropical Studies

The optimal SD identified here (July 20) differs from recommendations for other mung bean–growing regions in Ethiopia. For example, Kawte et al. [38] recommended late June to early July sowing for southern Ethiopia, reflecting the earlier onset of rains in that region. Similarly, studies in South Asia have reported optimal sowing windows in June–July, corresponding to the monsoon onset [11]. The later optimal SD in Western Ethiopia reflects the specific rainfall pattern of this location, where the main rains commence in late June to early July. This underscores the importance of location‐specific agronomic recommendations rather than broad extrapolation.

### 4.7. Integration of Biochar and Soil Amendments Literature

Beyond agronomic management, soil amendments such as biochar and plant‐growth–promoting rhizobacteria have shown promise for enhancing drought tolerance in grain legumes. For example, the combined application of biochar and arbuscular mycorrhizal fungi improved growth, root morphological traits, and soil biochemical properties in common bean under drought stress [53]. Similarly, biochar combined with salicylic acid has been demonstrated to alleviate salt stress in kidney beans in arid and semiarid regions [54]. While these approaches were not evaluated in the present study, they represent complementary strategies that could be integrated with optimized SD and spacing in future research.

### 4.8. Economic Analysis

A partial budget analysis using 2024/25 local prices (mung bean: 65 ETB/kg^−1^; NPS fertilizer: 28 ETB/kg^−1^; labor: 150 ETB person‐day^−1^) indicated that the July 20 × 25 cm treatment generated a net benefit of 124,500 ETB ha^−1^, compared with 28,700 ETB ha^−1^ for the farmer‐practice control (August 10 × 40 cm). The marginal rate of return was 417%, well above the 100% threshold conventionally used to define acceptable agronomic recommendations [55]. These results demonstrate the economic viability of the recommended treatment package.

### 4.9. Agronomic and Economic Implications for Practitioners

The practical implications of this research for smallholder farmers in Western Ethiopia and areas with comparable agroecologies are profound. The recommended package (July 20 sowing + 25 cm IRS) uses low‐cost, easily adoptable techniques that can more than double current farm‐level yields from ∼1000 kg/ha^−1^ to over 2200 kg/ha^−1^. This can directly translate into improved household food availability and a more than twofold increase in cash income from mung bean sales [14]. For extension agents, these findings provide a clear, research‐informed, and location‐specific recommendation to replace previous guesswork and generic advice, thereby accelerating the adoption of improved mung bean production practices.

Potential barriers to adoption include seed availability of the Rasa variety at the village level, labor constraints during the recommended sowing window, and the opportunity cost of allocating land to mung bean rather than to a cereal staple. Extension programs should address these constraints through seed multiplication schemes and demonstration plots.

### 4.10. Limitations and Future Research Directions

Several physiological mechanisms proposed to explain the observed treatment effects including light interception, source–sink relationships, and assimilate partitioning were not directly measured in this study. These explanations remain plausible hypotheses rather than demonstrated mechanisms. Future research should incorporate measurements of intercepted radiation, leaf gas exchange, and soil moisture dynamics to elucidate the underlying physiological basis of the SD × spacing interaction.

Furthermore, this study was conducted at a single location during one cropping season. Seasonal rainfall variability is a major determinant of mung bean performance in rain‐fed systems, and the specific sowing window and spacing recommendations should be validated across multiple years and locations before broad‐scale dissemination. Future trials should be conducted across at least three locations representing contrasting rainfall regimes (e.g., Uke, Nekemte, and Dembi Dolo) over three consecutive seasons to capture seasonal variability. Economic analysis (partial budget and marginal rate of return) of this treatment package versus farmers’ practice is also recommended to solidify the case for adoption.

## 5. Conclusions

This study provides the first empirical, location‐specific evidence on the optimal agronomic management of the mung bean variety Rasa in the rain‐fed lowland agroecology of Western Ethiopia. The results provide evidence that crop productivity in this environment is governed by a highly significant interaction between the timing of sowing and the density of the plant stand, meaning that managing these two factors independently will invariably lead to suboptimal outcomes.

Foremost, SD emerged as the dominant factor, significantly dictating the crop’s entire life cycle from the duration of phenological phases and growth rate to the formation of yield components and the final economic yield. A delay in sowing from July 20 to August 10 forcibly and substantially accelerated crop maturity, severely truncated the vegetative growth period, and precipitated a substantial decline in SY, thereby underscoring the prohibitive risk associated with late planting under rain‐fed conditions. The temperature and moisture gradients recorded during the experimental season with mean daily temperatures increasing from 21.5°C to 24.8°C and cumulative rainfall to flowering declining from 285 mm to 87 mm as sowing was delayed provide quantitative support for this phenological acceleration.

IRS exerted a critical and quantifiable influence on canopy architecture: Narrower spacings consistently generated a higher LAI, which was, in turn, strongly and positively correlated with enhanced biological and SY. Crucially, however, the ultimate effect of spacing on SY was conditional upon the date of sowing. The synergistic combination that maximized SY achieving a notable 2245.0 kg/ha^−1^ was an early SD of July 20 coupled with a moderate IRS of 25 cm. This optimal treatment succeeded by effectively balancing a prolonged, favorable growing season with a plant density that maximized total biomass accumulation and ensured efficient partitioning of photosynthates to the economic sink. This combination achieved a functional equilibrium between interplant competition and canopy resource capture: The 25‐cm row spacing allowed sufficient plant density to achieve a high LAI (0.32) and BY, while avoiding the intense intraspecific competition at 20 cm that reduced pods per plant and seeds per pod.

Finally, the path to high yield in this agroecology was confirmed to be source‐limited, as SY was most strongly and positively correlated with total BY (*r* = 0.70, *p* < 0.01) and LAI (*r* = 0.80, *p* < 0.01), the primary drivers of photosynthetic capacity. These findings collectively establish that the strategic integration of an early sowing window with a precisely calibrated IRS is a promising agronomic strategy for improving mung bean productivity in Western Ethiopia.

### 5.1. Acknowledgment of Study Limitations

These conclusions are drawn from a single location and one cropping season. Seasonal rainfall variability is a major determinant of mung bean performance in rain‐fed systems, and the specific sowing window and spacing recommendations should be validated across multiple years and locations before broad‐scale dissemination. Several physiological mechanisms proposed to explain the observed treatment effects including light interception, source–sink relationships, and assimilate partitioning were not directly measured in this study. These explanations remain plausible hypotheses rather than demonstrated mechanisms. Future research should incorporate measurements of intercepted radiation, leaf gas exchange, and soil moisture dynamics to elucidate the underlying physiological basis of the SD × spacing interaction. Additionally, no site‐specific soil test‐based fertilizer prescription was available for this location; this represents a further limitation of the study.

### 5.2. Target Audience and Economic Viability

The primary target audience for these recommendations is smallholder farmers in the rain‐fed lowland agroecologies of Western Ethiopia. A partial budget analysis using 2024/25 local prices indicated that the July 20 × 25 cm treatment generated a net benefit of 124,500 ETB ha^−1^, compared with 28,700 ETB ha^−1^ for the farmer‐practice control (August 10 × 40 cm). The marginal rate of return was 417%, well above the 100% threshold conventionally used to define acceptable agronomic recommendations. These results demonstrate the economic viability of the recommended treatment package, which uses low‐cost, easily adoptable techniques that can more than double current farm‐level yields from ∼1000 kg/ha^−1^ to over 2200 kg/ha^−1^. This can directly translate into improved household food availability and a more than twofold increase in cash income from mung bean sales.

### 5.3. Recommendations for Research, Extension, and Policy

For immediate application: The Guto Gida District Agriculture Office and relevant NGOs should actively promote and demonstrate the use of a July 20 SD and a 25‐cm IRS for the Rasa mung bean variety to smallholder farmers in the Uke area and similar lowland agroecologies of Western Oromia. Extension programs should address potential barriers to adoption including seed availability of the Rasa variety at the village level, labor constraints during the recommended sowing window, and the opportunity cost of allocating land to mung bean rather than to a cereal staple through seed multiplication schemes and demonstration plots.

For future research: Future trials should be conducted across at least three locations representing contrasting rainfall regimes (e.g., Uke, Nekemte, and Dembi Dolo) over three consecutive seasons to capture seasonal variability and validate the stability of this recommendation. Economic analysis (partial budget and marginal rate of return) of this treatment package versus farmers’ practice across different price scenarios is also recommended to solidify the case for adoption. Research should also incorporate measurements of intercepted radiation, leaf gas exchange, and soil moisture dynamics to elucidate the underlying physiological basis of the SD × spacing interaction. Furthermore, the integration of soil amendments such as biochar and arbuscular mycorrhizal fungi, which have shown promise for enhancing drought tolerance in other grain legumes could be explored as complementary strategies alongside optimized SD and spacing.

For breeding programs: Breeding programs targeting rain‐fed lowland environments should prioritize early canopy vigor and the ability to attain high LAI rapidly after emergence, as these traits are strongly associated with BY and SY in this agroecology. Additionally, traits associated with successful early sowing such as a long vegetative period, high LAI, and high BY potential should be prioritized for rain‐fed, terminal‐drought‐prone environments.

In summary, this study provides evidence that the yield of the Rasa mung bean variety in the study area is significantly influenced by an interactive effect of SD and plant spacing. The combination of a July 20 SD and a 25‐cm IRS is recommended for optimal yield and yield components under rain‐fed conditions in Western Ethiopia and similar agroecologies, pending multiseason and multilocation validation. These findings provide a research‐informed, location‐specific agronomic recommendation that can empower smallholder farmers to significantly improve mung bean productivity and contribute to regional food and income security.

## Author Contributions

Conceptualization, Birki Binalfew and Usman Mohammed Ali; methodology, Birki Binalfew, Temesgen Fasika, and Usman Mohammed Ali; software, Birki Binalfew; validation, Birki Binalfew, Temesgen Fasika, and Usman Mohammed Ali; formal analysis, Birki Binalfew; investigation, Birki Binalfew, Temesgen Fasika, and Usman Mohammed Ali; resources, Usman Mohammed Ali; data curation, Birki Binalfew; writing–original draft preparation, Birki Binalfew; writing–review and editing, Birki Binalfew, Temesgen Fasika, and Usman Mohammed Ali; visualization, Birki Binalfew and Temesgen Fasika; supervision, Usman Mohammed Ali.

## Funding

This research received no external funding.

## Ethics Statement

This study did not involve human participants, animals, or any sensitive ethical issues.

## Consent

The authors have nothing to report.

## Conflicts of Interest

The authors declare no conflicts of interest.

## Data Availability

The raw data supporting the conclusions of this article will be made available by the corresponding author upon reasonable request.
